# Multicellular 3D Neurovascular Unit Model for Assessing Hypoxia and Neuroinflammation Induced Blood-Brain Barrier Dysfunction

**DOI:** 10.1038/s41598-020-66487-8

**Published:** 2020-06-17

**Authors:** Goodwell Nzou, Robert T. Wicks, Nicole R. VanOstrand, Gehad A. Mekky, Stephanie A. Seale, Aya EL-Taibany, Elizabeth E. Wicks, Carl M. Nechtman, Eric J. Marotte, Vishruti S. Makani, Sean V. Murphy, M. C. Seeds, John D. Jackson, Anthony J. Atala

**Affiliations:** 1 1Wake Forest Institute for Regenerative Medicine, Wake Forest School of Medicine, Winston-Salem, NC 27101 USA; 2 Department of Neurology and Neurological Surgery, Wake Forest Baptist Medical Center, Winston-Salem, NC 27157 USA; 3 Zoology Department, Faculty of Science, Zagazig University, Zagazig, Egypt

**Keywords:** Blood-brain barrier, Stroke

## Abstract

The blood-brain barrier (BBB) is a dynamic component of the brain-vascular interface that maintains brain homeostasis and regulates solute permeability into brain tissue. The expression of tight junction proteins between adjacent endothelial cells and the presence of efflux proteins prevents entry of foreign substances into the brain parenchyma. BBB dysfunction, however, is evident in many neurological disorders including ischemic stroke, trauma, and chronic neurodegenerative diseases. Currently, major contributors to BBB dysfunction are not well understood. Here, we employed a multicellular 3D neurovascular unit organoid containing human brain microvascular endothelial cells, pericytes, astrocytes, microglia, oligodendrocytes and neurons to model the effects of hypoxia and neuroinflammation on BBB function. Organoids were cultured in hypoxic chamber with 0.1% O_2_ for 24 hours. Organoids cultured under this hypoxic condition showed increased permeability, pro-inflammatory cytokine production, and increased oxidative stress. The anti-inflammatory agents, secoisolariciresinol diglucoside and 2-arachidonoyl glycerol, demonstrated protection by reducing inflammatory cytokine levels in the organoids under hypoxic conditions. Through the assessment of a free radical scavenger and an anti-inflammatory endocannabinoid, we hereby report the utility of the model in drug development for drug candidates that may reduce the effects of ROS and inflammation under disease conditions. This 3D organoid model recapitulates characteristics of BBB dysfunction under hypoxic physiological conditions and when exposed to exogenous neuroinflammatory mediators and hence may have potential in disease modeling and therapeutic development.

## Introduction

The blood-brain barrier (BBB) comprises the physical and enzymatic barrier between the brain and the bloodstream. Comprised primarily of specialized non-fenestrated endothelial cells along the vascular wall, pericytes, and astrocyte foot processes, this BBB architecture controls the selective transport of the brain’s supply of nutrients and hormones, as well as the removal of metabolic wastes, foreign substances, and excess neurotransmitters. The critical role played by the BBB in maintaining homeostasis within the central nervous system (CNS) makes it clear why BBB breakdown is evident in many neurological disorders. During ischemic stroke, in particular BBB breakdown leads to edema and hemorrhage^1,2^. These pathologic consequences worsen secondary brain injury and significantly contribute to cognitive impairment. Hence pathological effects of hypoxia on BBB function may be critical in understanding strategic therapeutic targets for BBB maintenance and recovery during and after neurologic injury.

Major hurdles to the development of novel molecular therapies for ischemic stroke include, but are not limited to; the lack of an agreed upon ischemic stroke *in vitro* model for pre-clinical drug screening, and the fundamental differences in BBB organization and architecture between humans and the most common animal models. Current *in vitro* models of ischemic stroke differ in cell types utilized, human vs. rodent, in structural design, monoculture vs. co-culture, and co-culture in monolayer form vs. layered form^3^.

Clinical treatments for acute stroke include tissue plasminogen activator (tPA) administration^4–6^ and thrombectomy^7,8^. In patients with acute ischemic stroke, thrombectomy and tPA therapy allow for salvage of penumbral or “at risk” central nervous system (CNS) tissue. However, despite good revascularization and being within the therapeutic time window not all patients benefit from thrombectomy or tPA therapy. In penumbral or “at risk” CNS tissue, physiological changes, such as hypoxia and inflammation, within the neurovascular unit alter the normal function and lead to death of penumbral tissue even after subsequent revascularization. Given that there are many patients that do not benefit from thrombectomy or tPA therapy, development of a novel therapy that would protect penumbral or “at risk” tissue is therefore needed.

Inflammatory tissue injury is very common in many neurological disorders including stroke and is believed to be mediated by reactive metabolites that include reactive oxygen species (ROS), reactive nitrogen species, and reactive sulfur species^9–11^. These reactive species cause deleterious complications such as lipid peroxidation that can cause damage to cellular membrane and trigger second messengers that lead to apoptosis. An *in vitro* model that can recapitulate both the changes to the BBB architecture and the inflammatory stress response that occurs in response to hypoxia is critical to defining new therapeutic targets for mitigating the resulting ongoing neurologic injury.

We have recently developed a six cell-type neurovascular unit human organoid model containing brain microvascular endothelial cells, pericytes, astrocytes, oligodendrocytes, microglia, and neurons for use in neurotoxicity screening and disease modeling^12^. Our previous findings show endothelial cells coating the outer sphere of the organoids. We also reported that these endothelial cells express functional tight junctions that reduced paracellular transport of labeled IgG and the neurotoxin MPP+ (1-methyl-4-phenylpyridinium). Six cell type organoids had reduced IgG permeability compared to organoids containing endothelial cells, pericytes, astrocytes only^12^. This indicated the importance of multiple cell interactions in the maintenance and function of the BBB in the neurovascular unit as described in Nzou *et al*.^13^. We then evaluated and observed disrupted tight junction markers in organoids cultured under hypoxic condition^12^. Here, we utilized this human cell-based 3D *in vitro* model to measure the effects of hypoxia on BBB structure and function. During stroke the immediate tissue around the occluded vessel (the ischemic core) die within a short period due to a dearth of oxygen and nutrients. However, the tissue around the ischemic core, called the ischemic penumbra, have access to minimum levels of oxygen and nutrients secondary to nearby collateral vasculature. This is the region that is of interest when considering rescuing either thrombotic or embolic stroke. Under only low oxygen supply, we attempted to create similar physiologic conditions in order to evaluate the effect of hypoxia on BBB structure and function.

We evaluated the expression levels of proteins critical in BBB maintenance, basement membrane proteins, tight junction proteins, and BBB transport proteins. We also assessed the secretion and effect of inflammatory mediators under hypoxic condition. Our results showed significant change in chemokines and cytokines, heat shock proteins, transport proteins, tight junctions and basement membrane protein expression under hypoxia. These changes may contribute to BBB dysfunction under hypoxic conditions. Through the assessment of a free radical scavenger and an anti-inflammatory endocannabinoid, we hereby report the utility of the model in drug development for drug candidates that may reduce the effects of ROS and inflammation under disease conditions. This human cortex organoid placed within a hypoxic environment mimics normal physiologic response and forms the basis for a promising disease model that could potentially be implemented as an initial *in vitro* drug screening tool in the evaluation of novel therapeutics.

## Results

### Hypoxia induced IgG and albumin permeability

**Figure 1:**
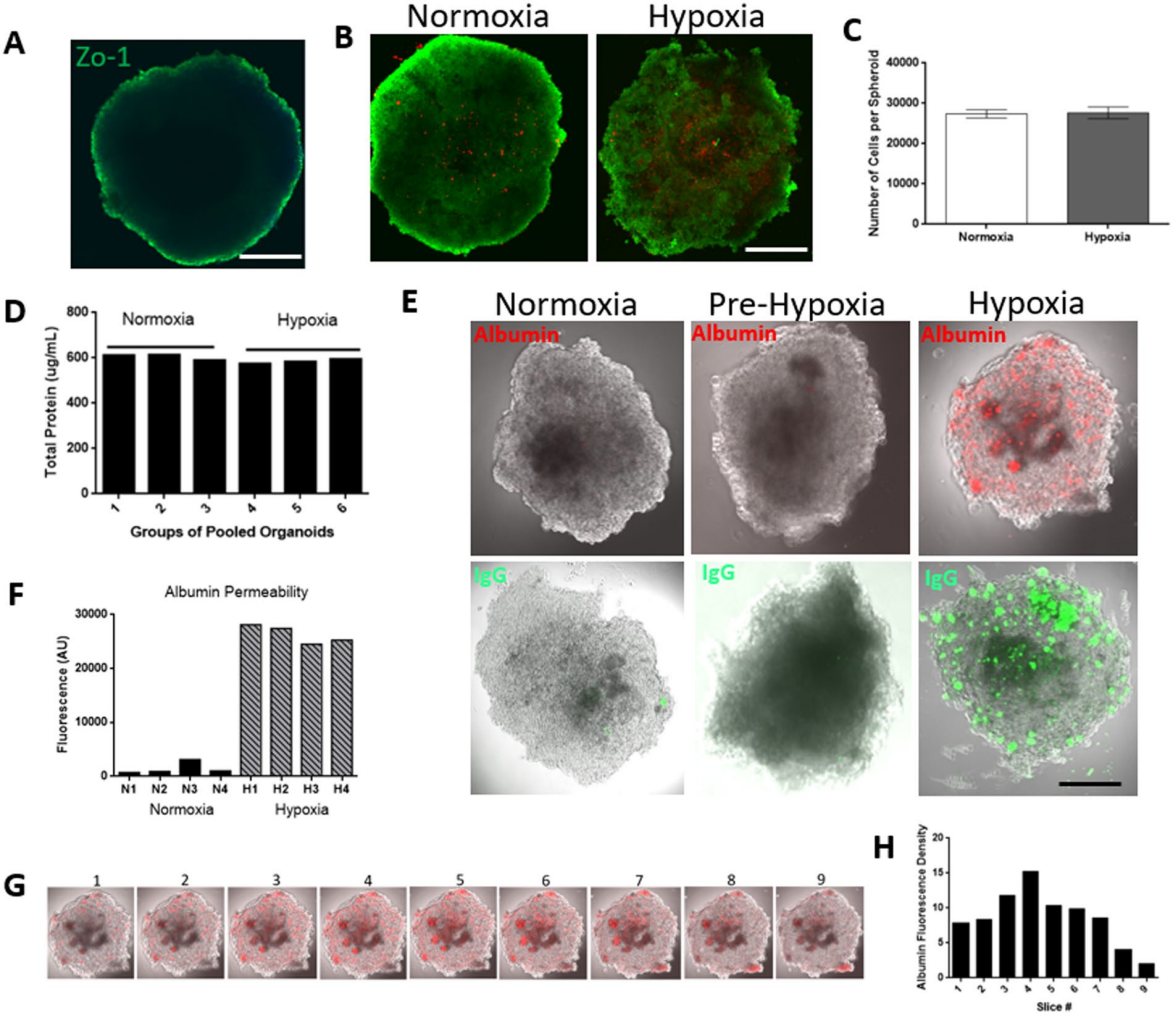
Hypoxia induced permeability and cell death. (**A**) Zo-1 staining around the organoid showing complete BBB coverage around the organoids. Cell viability was assessed using calcein AM (green- live cells) and ethidium homodimer 1 (red- dead cells) (**B**). The number of cells per organoid under both normoxic and hypoxic condition was determined by pooling 24 organoids into an eppendorf tube. Cell suspensions were obtained by dissociating the organoids with dispase. The cells were subsequently counted, and the data is shown in (**C**). Protein levels for six groups containing 80 organoids each were obtained from six randomly chosen plates and total protein levels were determined by BCA assay in (**D**). In (**E**), 10 organoids were used in each group and 5 randomly chosen organoids were imaged. The results show increased permeability of labeled albumin (red) and FITC labeled IgG (green) into organoids post hypoxic culture conditions compared to no penetration of these proteins under normoxic conditions. Pre-hypoxia represents organoids from the same batch that were pooled and assessed for albumin and IgG permeability prior to culturing under hypoxic condition. Images shown are middle slices of the z-stack confocal image. Fluorescence of albumin was quantified in 4 organoids under normoxic condition and post hypoxia as illustrated in F. (**G**) Shows the individual slices across the organoid and (**H**) the quantification of fluorescent albumin density from (**E**) within the organoid of each slice. Student T-Test, two tailed hypotheses, *P < 0.05. Data represented as mean $\pm \,{\rm{SEM}}.$ Scale bars 200 μm.

Previous results show that the staged assembly method allow for a complete coverage of endothelial cells on the outer sphere. We have shown that these endothelial cells form a functional BBB^12^. Here, we only show ZO-1 staining (Fig. 1A) to highlight the complete coverage of endothelial cells on the organoids. In the examination of cell viability, we observed significant increase in cell death in cores of organoids cultured under hypoxic condition compared with normoxia-cultured counterparts (Fig. 1B). There was no detectable cell death on the surface of organoids under hypoxia, however, the outer layer was more disorganized compared to the outer layers of organoids under normoxia (Fig. 1B). We evaluated the number of cells per organoid under hypoxia and normoxic condition and observed a non-significant decrease in normoxic organoids. Although the cell count varied within groups (Fig. 1C), organoids from the same batch under normoxic and hypoxic conditions were qualitatively similar in size (Fig. 1B,E) and cell lysates had approximately equal total protein levels from both groups (Fig. 1D). The variety in cell counts and differences in these results could be attributed to an incomplete dissociation of organoids. We previously demonstrated that 24 hours of hypoxic stress disrupts the distribution of tight junctions at the BBB^12^. Since tight junctions play a major role in preventing free paracellular transport of blood born components^14–17^, we anticipated increased permeability of large molecules upon culturing our organoids under hypoxic conditions. Thus, we evaluated the ability of FITC-labeled IgG and FITC-labeled albumin to cross the BBB in organoids cultured under hypoxic vs. normoxic conditions. Qualitatively, greater amounts of IgG and albumin crossed the BBB in organoids that underwent hypoxic treatment than in those under normoxia (Fig. 1E). BBB integrity assessment prior to hypoxia incubation revealed an intact and functional BBB shown by no albumin or IgG penetration into the pre-hypoxia organoids (Fig. 1E). BBB integrity in individual organoids was assessed by quantifying albumin fluorescence in organoids cultured under normoxia and hypoxia. The results indicate variability in albumin penetration within each group, highlighting varied BBB integrity in organoids from the same batch cultured under the same conditions (Fig. 1F). This may be attributed to variations in neurovascular unit cell localization which may have a direct impact on BBB integrity depending on the proximity of supporting cells to BBB forming human brain microvascular endothelial cells. Further studies are needed to elucidate localization and the subsequent effects on BBB integrity. To evaluate albumin distribution within organoids cultured under hypoxia, albumin fluorescence was quantified from individual slices (Fig. 1G). We observed high fluorescence density in middle slices for albumin (Fig. 1H) and IgG (not shown).

### Protein expression was altered by hypoxia

**Figure 2:**
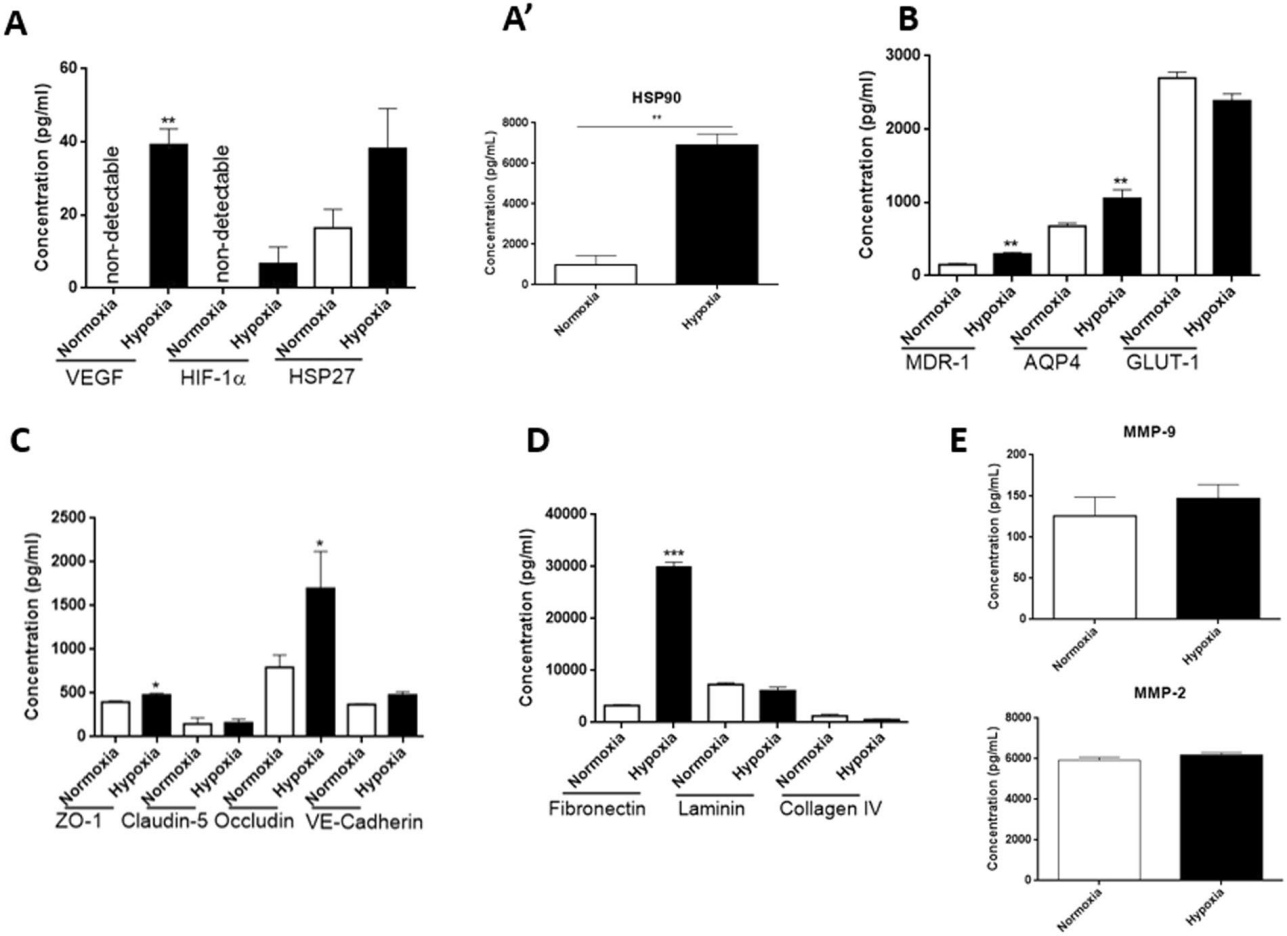
Hypoxic stress induces alterations in BBB transport, junctional and basement membrane protein levels in organoids. Two groups of 80 organoids each were pooled and cultured under hypoxic conditions and normoxic culture conditions for comparison. In **A**, total protein was extracted from 20 spheroid and the total extract was then pipetted into 6 wells for the ELISA. The expression of hypoxia induced proteins was only significantly higher under hypoxic condition compared to normoxic condition for VEGF. There was no significant difference in the expression of HIF-α and HSP27 between the two conditions (**A**) and HSP90α expression was increased under hypoxic condition (**A’**). Total protein expression of MDR-1 and AQP4 was significantly increase under hypoxic condition compared to normoxic condition **(B**). Total expression of GLUT-1 expression was not significantly different between culture conditions. Claudin-5 and VE-Cadherin expression was not significantly higher under hypoxic condition (**C**). Expression of other tight junction proteins Occludin and ZO-1 were significantly higher under hypoxic condition compared to normoxic condition (**C**). The expression of basement membrane protein was also evaluated, and Fibronectin had a significantly higher expression under hypoxic condition, while Laminin and Collagen IV expression was lower in organoids cultured under hypoxic conditions (**D**). In (**E**), protein expression of MMP-9 (upper panel) in hypoxic condition was not different when compared to normoxic condition. Similarly, MMP-2 (lower panel) expression was not significantly higher under hypoxic conditions compared to normoxic condition Student T-Test, two tailed hypothesis, n = 6 wells per condition, *P < 0.05, **P < 0.01, ***P < 0.001. Data represented as mean $\pm {\rm{SEM}}$.

Hypoxia-induced vascular endothelial growth factor (VEGF) production may have angiogenic effects in the sprouting of new vessels that provide additional nutrients to starved regions^18^. However, VEGF also is known to disrupt tissue endothelial barrier functions^19^. Consequently, we evaluated the expression of VEGF under hypoxic conditions. Levels of VEGF were significantly increased in organoids cultured under hypoxia (39.2 pg/ml, P = 0.0022) compared to their counterparts that were grown under normoxic condition (undetectable, Fig. 2A). We identified higher levels of hypoxia-induced factor 1-alpha (HIF-1α) under hypoxic condition, which also plays a role in vascular permeability under disease conditions^20,21^ (Fig. 2A). Heat shock proteins are upregulated under conditions of stress to stabilize new proteins by promoting their proper folding, or to assist in refolding proteins that may have been damaged by hypoxia-induced stress^22^. To that end, we found an upwards trend of heat shock proteins 27 (HSP27) levels (Fig. 2A) and significantly more expression of HSP90 (Fig. 2A’) in brain organoids that underwent hypoxic conditions

Transport proteins at the BBB level crucially regulate the transport of some blood-borne substances to the parenchymal tissue. We identified the effect of hypoxia on the expression of three major transport proteins at the BBB. Total protein levels of both multidrug resistance protein 1 (MDR-1) and aquaporin 4 (AQP4) were significantly increased in organoids cultured under hypoxic conditions (295.6 pg/ml and 1054.1 pg/ml) than in those cultured under normoxic condition (151.2 pg/ml, P = 0.0043, and 675.7 pg/ml, P = 0.0043), respectively (Fig. 2B). However, the expression of the glucose transporter was not significantly different, (2.4 ng/ml, P = 0.093) compared to normoxia (2.7 ng/ml) (Fig. 2B).

Also critical to the function of the BBB, are the junctional proteins that prevent free diffusion of molecules through the BBB. To understand the effect of hypoxia on the expression of junctional proteins, we measured their total protein expression in our organoids. Zona occludin 1 (ZO-1) and occludin expression were increased under hypoxic (480 pg/ml and 1.7 ng/ml) compared to normoxic conditions (399.5 pg/ml, P = 0.0023, and 0.8 ng/ml, P = 0.026); however, Claudin 5 and VE-cadherin was not significantly changed by hypoxia (Fig. 2C).

The basement membrane regulates the structural integrity of the BBB; however, the proteins that comprise the basement membrane vary under disease conditions^23^. In this study, we quantified the amounts of fibronectin, laminin 1, and collagen type 4. Fibronectin expression was significantly increased in organoids cultured under hypoxic conditions (138 pg/ml normoxic vs. 2567 pg/ml hypoxia, P = 0.0007), while laminin (633.7 pg/ml normoxia vs. 458.8 pg/ml hypoxic, P = 0.0593) and collagen (1.3 ng/ml normoxia vs. 0.3 ng/ml hypoxia, P = 0.0379) trended lower under hypoxic conditions (Fig. 2D). The expression levels of matrix metalloproteinases, MMP-9 and MMP-2 were not significantly higher (126 pg/ml normoxia vs. 149 pg/ml hypoxia) and (5.9 ng/ml normoxia vs. 6.2 ng/ml hypoxia), respectively (Fig. 2E).

### Hypoxia increased oxidative stress

**Figure 3:**
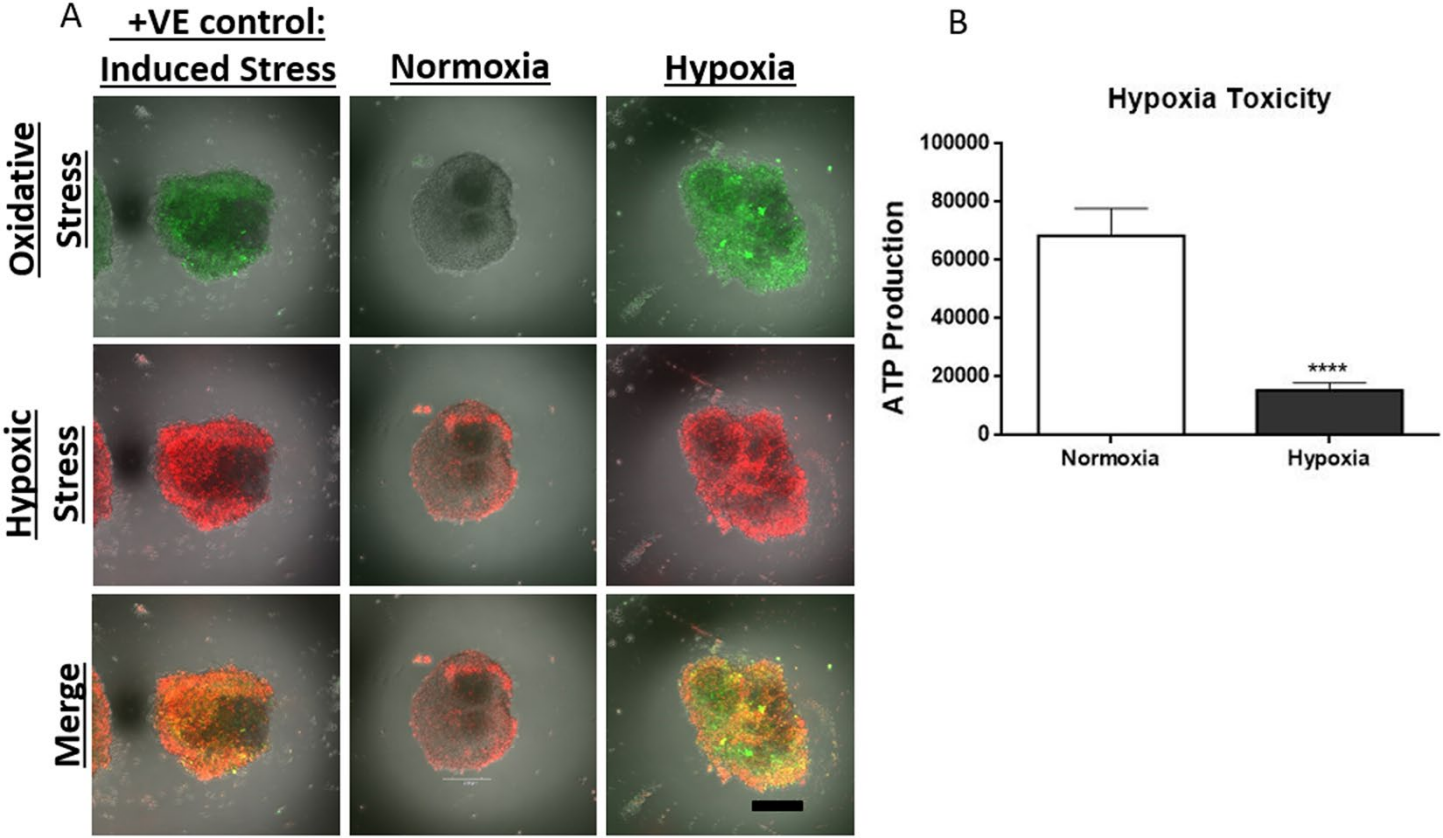
Hypoxia induces oxidative stress. In (**A**), 10 organoids in each in each of the three group were pooled and cultured under hypoxic conditions and normoxic culture conditions. The positive control was prepared by treating the normoxic organoids with oxidative stress inducer, Pyocyanin and hypoxic inducer, Deferoxamine. All groups were then incubated in hypoxic and oxidative stress detection reagent that reacts with oxidative stress mediators to produce a fluorescent product. Images were obtained using a confocal microscope. Qualitative results show more hypoxic (Red) and oxidative stress (Green) in organoids cultured under hypoxia. In (**B**), metabolic activity was assessed by measuring ATP production. There was a significant decrease in ATP production in hypoxic organoids. Student T-Test, two tailed hypothesis, n = 40 organoids per group in B, ****P < 0.0001. Data represented as mean $\pm \,{\rm{SEM}}$. Scale bars 250 μm.

Decreased partial pressure of oxygen has been associated with antioxidant imbalance and changes in mitochondrial oxidative phosphorylation that lead to increased amounts of reactive oxygen and nitrogen species (RONS) and subsequent oxidative damage to lipids, proteins, and DNA^24^. To characterize the effect of hypoxia in our organoids, organoids cultured under normoxic and hypoxic conditions were incubated with dyes containing functional moieties that react with oxidative stress mediators. Specifically, the hypoxic reagent dye contains non-fluorescent aromatic compound with a nitro group. The high nitroreductase activity in hypoxic cells converts the nitro moiety in a series of reactions to an amino group to produce a fluorescent molecule^25^. Similarly, the oxidative stress reagent is a cell permeable non-fluorescent dye that reacts with wide range of reactive species, such as hydrogen peroxide, peroxynitrite, and hydroxyl radicals, yielding a fluorescent product indicating the presence of RONS. Qualitative analysis shows higher amounts of oxidative stress in organoids cultured under the hypoxic condition (Fig. 3A). Since oxidative stress has damaging consequences on mitochondrial function^26^, we measured ATP production in our organoids to evaluate metabolic activity. ATP production was significantly decreased in organoids cultured under hypoxic condition (Fig. 3B).

### Hypoxia induced the production of proinflammatory cytokines

**Figure 4:**
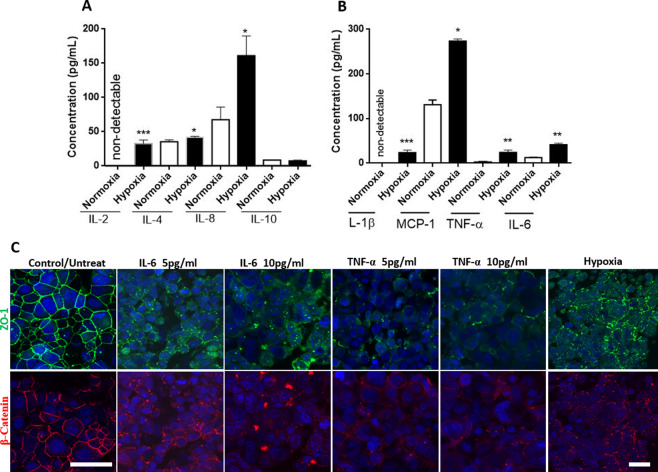
Cytokine production and the effect of exogenous cytokines. In A and B, two groups of 50 organoids each were pooled and cultured under hypoxic conditions and normoxic culture conditions for comparison. Total protein extract from 50 organoids was then pipetted into 6 wells for the ELISA. There was significantly higher secretion of IL-2, IL-4, and IL-8 under hypoxic condition compared to normoxic condition, however, IL-10 secretion was not different between the two culture conditions (**A**). The expression and secretion of inflammatory cytokines IL-1β, TNF-α, and IL-6 secretion was significantly elevated under hypoxic condition compared to normoxic conditions (**B**). MCP-1 secretion was significantly higher under hypoxic condition compared to normoxic conditions. In **C**, we highlight the effect of exogenous cytokines on the distribution of tight junction at the Blood-Brain Barrier. Normal distribution of the tight junctions is evident under normoxic condition while the tight junctions are disrupted when treated with both IL-6 and TNF-α at low or high concentrations. Similar disruption of the tight junction was also observed in organoids cultured under hypoxic condition. Student T-Test, two tailed hypothesis, n = 6 wells for A and B *P < 0.05, **P < 0.01, ***P < 0.001. Data represented as mean $\pm \,{\rm{SEM}}$. Scale bars (control 40 μm, treated and hypoxia 40 μm).

Neuroinflammation is implicated in many neurological diseases and disorders^27–30^. Cellular and molecular magnetic resonance imaging revealed neuroinflammation in post-stroke studies^31^. The molecular mechanistic causes of neuroinflammation are not well understood. However, it is thought that oxidative stress under hypoxic conditions activates glial cells, specifically the microglia^32^. Glial activation may result in the production of proinflammatory cytokines and chemokines that recruit peripheral leucocytes and subsequently disrupt the BBB^33,34^. We determined the expression of both pro-inflammatory and anti-inflammatory cytokines, which augment neuroinflammation together with complement and free radicals^34^. Most of pro-inflammatory cytokines and chemokines we measured (IL-2, MCP-1, IL-4, IL1β, TNF-α, and IL-6) were significantly upregulated under hypoxic condition (Fig. 4A-B). Interleukin (IL)-4, which can have anti-inflammatory effects through its Th-2 and alternative macrophage activities, was also significantly higher under hypoxic conditions than under normoxic conditions, although anti-inflammatory IL-10 secretion was not changed (Fig. 4A).

### Exogenous cytokines directly impacted BBB integrity

**Figure 5:**
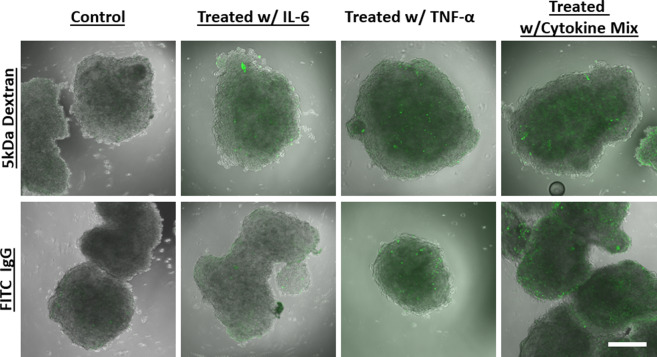
Exogenous cytokines increased BBB permeability. Organoids were incubated with IL-6 only, TNF-α only, or a combination of cytokines (IL-2, IL-6, VEGF, and TNF-α) for 12 hours. BBB permeability was assessed using a 5 kDa FITC dextran and FITC labeled IgG. The concentration for each of cytokines used was 5 ng/ml. The dextran molecule traversed across the BBB in organoids treated with cytokines. However, IL-6 alone did not induce BBB permeability. Scale bars 300 μm, the experiment was repeated twice. Images shown are middle slices of the z-stack confocal image.

Increased cytokine levels have a profound effect on the BBB through the induction of endothelial cells to produce prostaglandins, nitric oxide, free radicals, and additional chemokines^35,36^. Furthermore, patients with neurodegenerative diseases such as ALS^37^, Alzheimer’s disease^38^, Parkinson’s disease^39^, as well as survivors of stroke^40^ have shown to have increased cytokine levels. The direct effect of circulating cytokines on BBB dysfunction, however, has not been evaluated. To determine the effect of circulating cytokines on the BBB we incubated our organoids with the exogenous cytokines IL-6 and TNF-α for 24 hours. Our results show that these exogenous cytokines disrupted the distribution of tight junctions compared to untreated organoids (Fig. 4C). The disorganized tight junction markers are consistent with our previous observations of organoids cultured under hypoxic conditions^12^. Further studies assessing BBB permeability showed high permeability of FITC dextran in organoids treated with IL-6 or TNF-α and in organoids that were treated with a mix of cytokines (IL-2, IL-6, TNF- α, and VEGF, Fig. 5). FITC labeled IgG permeability was visually higher in organoids treated with TNF-α or the cytokine mix compared to organoids treated with IL-6 only (Fig. 5).

### Free radical scavenging decreased metabolic oxidative stress and reduced secretion of pro-inflammatory cytokines

**Figure 6:**
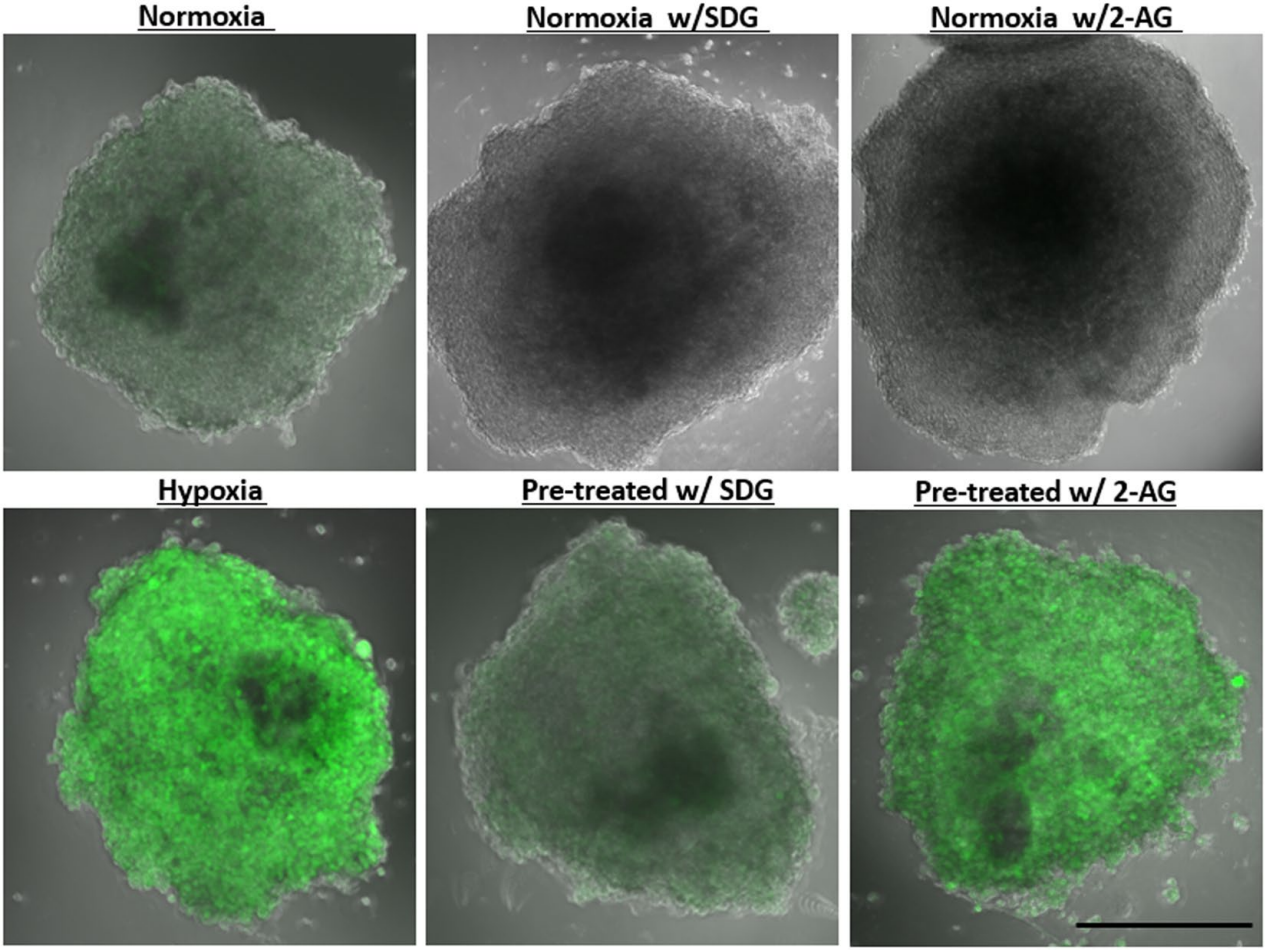
Treatment with Secoisolariciresinol diglucoside (SDG) decreased metabolic oxidative stress. Ten organoids were either treated with 50 µM SDG or 2- arachidonoyl glycerol 2-AG for 48 hrs prior to culturing in hypoxic chamber. Oxidative stress was then measured using oxidative stress reagent that produce a fluorescent product when it reacts with oxidative stress mediators. Four organoids from each group were then imaged using a scanning confocal microscope. SDG treatment reduced metabolic oxidative stress compared to organoids that were not treated prior to hypoxic incubation. Scale bars 300 μm.

**Figure 7:**
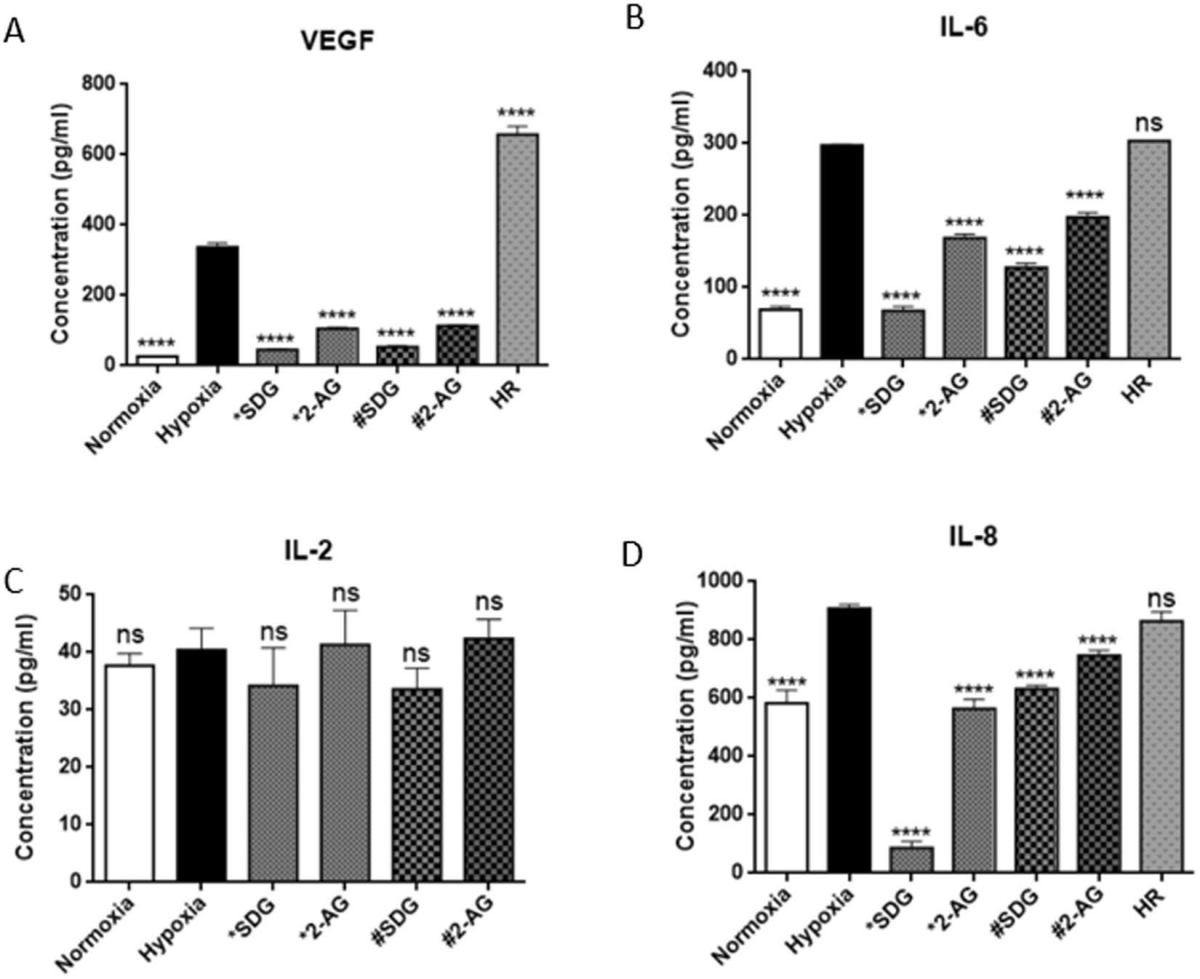
Treatment with Secoisolariciresinol diglycoside (SDG) and 2-Arachidonyl glycerol (2-AG) has an effect on cytokine production. Groups of 80 organoids each were pooled treated for 48 hours prior to culturing under hypoxic condition (*), treated during hypoxic condition only (**#**), or were non-treated, cultured under hypoxic conditions and then re-oxygenated under normoxic conditions for 24 hours (**HR)**. Total protein extract from 80 organoids was then added to 6 well for the ELISA. Expression of VEGF was significantly increased under hypoxic condition compared to normoxic condition (**A**). Preliminary experiments to assess effects of SDG and 2-AG on the expression of VEGF shows a significant decrease when treated with 2-AG while SDG elicited not significant decrease in VEGF expression when compared to hypoxic condition (**A**). There was a slight increase in the secretion on IL-6 under hypoxic condition compared to normoxic condition. Treatment with SDG lowered the secretion of the cytokine and 2-AG treatment resulted in a significant decrease compared to hypoxic condition (**B**). Re-oxygenated organoids secreted higher amounts of IL-6 compared to hypoxic condition (**B**). There was no significant difference in IL-2 secretion in organoids under hypoxia compared to those under normoxia. Furthermore, the treatments did not have a significantly different profile compared to organoids under hypoxic conditions (**C**). IL-8 secretion under hypoxia was significantly increased compared to normoxia. Pretreatment with both SDG and 2-AG resulted in reduction in IL-8 secretion while reoxygenation did not have an effect (**D**). One-way ANOVA multiple comparison to hypoxia was used to analyze the data. n = 6 wells, ***P < 0.001, ****P < 0.0001. Data represented as mean $\pm \,{\rm{SEM}}$.

An antioxidant free radical-scavenging drug 4-hydroxy-2,2,6,6-tetramethylpiperidine-*N*-oxyl (TEMPOL) with neuroprotective properties^41^ showed that oxidative stress may be a therapeutic target. At the BBB level, TEMPOL preserved occludin localization and prevented the collapse of occludin oligomeric assemblies in animals subjected to hypoxia and reoxygenation^41^. Here, we investigated the effects of another molecule; secoisolariciresinol diglucoside (SDG), a major lignan found in flaxseeds that has been hypothesized to have beneficial effects against diabetes, tumor progression, atherosclerosis, heart disease, oxidative stress, and inflammation^42^. SDG has also been shown to have BBB protective effects and anti-inflammatory properties due to its direct free radical scavenging activity^43–45^. Two experimental conditions were implemented: 1) 48 hours of pretreatment with SDG followed by placement in a hypoxic environment; 2) simultaneous treatment with SDG while the organoids were within a hypoxic environment for 24hrs. To measure oxidative stress, the organoids were incubated with non-fluorescent dye that reacts with oxidative mediators to produce a fluorescent product. Qualitative results show that pretreatment with SDG reduces oxidative stress mediators compared to untreated organoids under hypoxia (Fig. 6). Furthermore, we evaluated anti-inflammatory activity in our organoids by evaluating the effect of SDG on the secretion of pro-inflammatory cytokines. Our results show that pretreatment significantly reduced VEGF (Fig. 7A), IL-6 (Fig. 7B), and IL-8 (Fig. 7D) release into the supernatant. However, IL-2 (Fig. 7C) levels were not significantly reduced, neither by treating 48 hours prior to hypoxic incubation nor treating during hypoxia only.

### 2-Arachidonylglycerol (2-AG) had anti-inflammatory properties in hypoxia-induced neuroinflammation

The cannabinoid system has been shown to modulate cellular and molecular pathologies such as cell death, excitotoxicity, neuroinflammation, and cerebrovascular breakdown^46^. Specifically, 2-AG is a retrograde monoacylglycerol messenger in the endocannabinoid system that regulates plasticity and synaptic transmission^47–50^. *In vitro* studies with microglia and splenocytes have shown that 2-AG has anti-inflammatory effects in the form of reducing TNF-α and IL-2 release^51,52^. We first assessed and observed no effect on metabolic stress (RONS) when organoids were treated with 2-AG for 48 hours prior to culturing under hypoxia (Fig. 6). We also assessed the anti-inflammatory activity of 2-AG in hypoxia-induced inflammation, which has not previously been evaluated in a 3-dimensional organoid system. Protein quantities were determined using ELISA and we identified significantly lower VEGF (Fig. 7A), IL-6 (Fig. 7B), and IL-8 (Fig. 7D) secretion in organoids that were treated with 2-AG than in untreated controls that were cultured under hypoxia. There was no significant reduction in IL-2 (Fig. 7C) release.

### SDG and 2-AG treatment improved tight junction distribution

**Figure 8:**
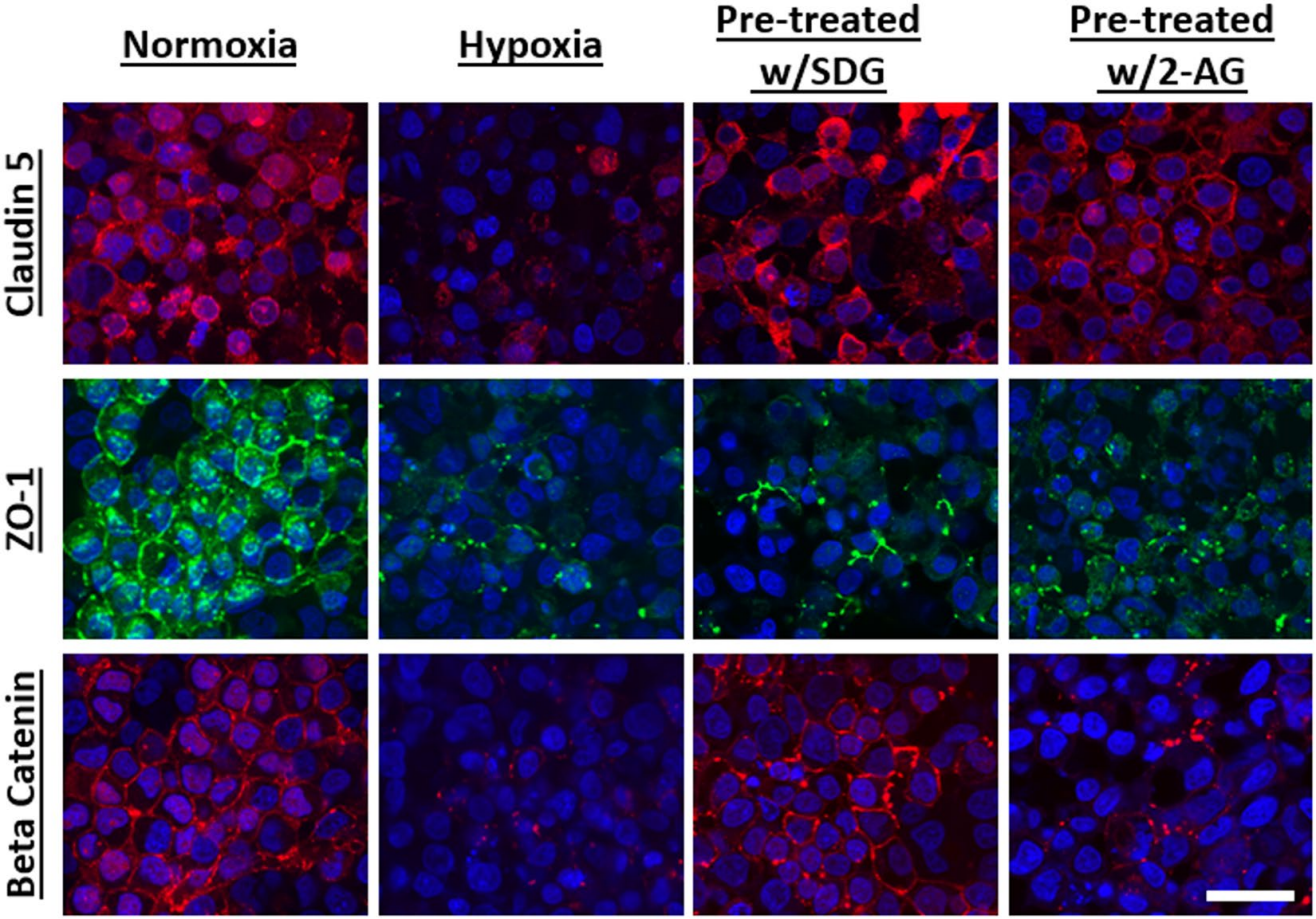
SDG and 2-AG improved tight junction distribution. Two groups of 20 organoids each were either treated with 50 µM SDG or 10 µM 2- arachidonoyl glycerol (2-AG) for 48 hours prior to culturing in hypoxic chamber. Then, another group of 20 untreated organoids and the treated groups were cultured under hypoxic condition for 24 hours. The organoids were then fixed and processed for immunohistochemistry staining and were subsequently imaged. Caludin-5 distribution and organization increased in organoids pre-treated with SDG and 2-AG. Beta catenin organization increased in organoids that were treated with SDG. Scale bars 300 μm.

At the BBB level, TEMPOL preserves occludin localization and prevents the collapse of occludin oligomeric assemblies in animals subjected to hypoxia and reoxygenation^41^. As highlighted above, SDG has free radical scavenging properties similar to TEMPOL. Here we assessed the effects of SDG on tight junction distribution. Results show improved organization of claudin-5 in organoids that were treated with SDG or 2-AG for 48 hours before culturing under hypoxic conditions (Fig. 8). There was no difference in the organizational distribution of ZO-1 in organoids that were treated compared to untreated organoids (Fig. 8). Additionally, β-catenin distribution was only maintained in organoids pre-treated with SDG.

## Conclusions and Discussion

Understanding how the BBB functions in healthy tissue and how its loss of function is linked to the progression of many neurological disorders is critical to identifying potential therapeutic strategies. Neuroimaging studies have revealed hippocampal BBB breakdown in individuals with mild cognitive impairment and early-stage Alzheimer’s disease^53^. Van de Haar *et al*. has also observed BBB dysfunction in the gray and white matter regions before brain atrophy or dementia^54,55^. Other neuroimaging studies have demonstrated BBB breakdown in Huntington’s disease, Alzheimer disease, Parkinson’s disease, amyotrophic lateral sclerosis, multiple sclerosis, HIV-1 associated dementia, chronic traumatic encephalopathy, stroke, and brain neoplasms^56–58^. The underlying mechanisms that result in BBB breakdown in many of these conditions are not well understood. However, it is believed that the loss of tight junction integrity occurs in a phasic manner as a consequence of many independent mechanisms including but not limited to transport protein channel dysregulation, inflammation, oxidative and nitrosative stress, and direct enzymatic injury^59^.

Hypoxic-ischemia results in acidosis, glutamate excitotoxicity, generation of reactive oxygen species, and oxidative stress. This is followed by cell death and prolonged periods of delayed inflammation^60,61^, which subsequently exacerbate the BBB breakdown in disease conditions. In this study, we identified increased BBB permeability in organoids that were treated under hypoxic conditions compared to those grown in normoxic conditions. Higher BBB permeability under hypoxic conditions may be a result of molecular and cellular dysfunctions secondary to cell death observed in organoids under hypoxic culture conditions. However, further studies are required to evaluate the extent of cell death in organoids and its effect on BBB function. Our studies also revealed altered transport protein expression under hypoxic conditions, specifically a higher expression of an efflux protein, MDR-1; this was expected given our observation of increased BBB permeability under hypoxic conditions. Higher permeability of xenobiotics across the BBB may lead to upregulation of MDR-1 expression in order to re-establish homeostasis. With the concentration of solutes increasing in the brain parenchyma, cells at the BBB level may upregulate the expression of AQP4 to increase the diluting water content in the interstitial tissue to reestablish equilibrium.

Tight junction expression serves as a BBB marker. We previously reported decreased tight junction levels under hypoxia. Here we also show a disruption of tight junctions under hypoxia as shown in Fig. 8. Interestingly, we observed increased expression in some tight junction proteins under hypoxic conditions compared with normoxic conditions, when assayed by ELISAs. This overall increase in tight junction protein levels did not correlate with the high BBB permeability we observed under hypoxic conditions. Other studies have shown that acute ischemic stroke in both human and rats increased peripheral circulating blood levels of tight junction proteins^62,63^. These studies showed significant increase in blood occludin levels and non-significant levels in blood claudin-5 levels^63^. These results demonstrate that fragments of cleaved tight junctions are released into circulating blood. The organoids used in the ELISA study to quantify tight junction levels were harvested and maintained in their native supernatant prior to addition of lysis buffer. The supernatant from organoids cultured under hypoxia may have contained cleaved fragments of tight junctions which resulted in overall increased levels in the ELISA study.

The expression of basement membrane proteins was also altered. We observed higher fibronectin expression under hypoxic conditions than under normoxic conditions. Fibronectin promotes angiogenesis^64^, and lack of oxygen within the organoid under hypoxic condition may induce angiogenic pathways that allow for oxygen and nutrient delivery to cells. In contrast, laminin (which has been shown to promote neuronal adhesion) and collagen levels were not significantly changed under these two conditions. Also, both MMP-2 and MMP-9 levels were not significantly increased under hypoxic conditions, indicating that BBB breakdown may not be due to autologous CNS upregulation of MMP-2 and MMP-9. Further enzymatic activity studies are needed to elucidate the effect of these enzymes on the BBB.

Understanding how inflammation causes BBB breakdown could provide therapeutic options for slowing neurologic disease progression by improving the integrity of the BBB^28,29,36,65^. We evaluated neuroinflammation in our organoids under hypoxic conditions by assessing the expression of inflammatory cytokines and chemokines and observed higher expression levels of pro-inflammatory mediators. This may indicate glial activation, specifically the astrocytes and microglia that are known to secrete pro-inflammatory cytokines under stress conditions^33,34^. This may be concomitant with increasing ROS in the organoid’s milieu^10^, and that in turn may exacerbate glial activation. We also observed higher expression levels of chemokines including IL-8 and MCP-1; these chemokines establish a gradient that recruits peripheral leukocytes to the site of the lesion^66,67^. Even though levels of some chemokines were not reliably significant between experiments, we observed consistent trends between experiments. The mechanism through which these chemokines cause BBB dysfunction in neurodegenerative disorders is not well characterized, but chemokine activity is observed in multiple sclerosis where peripheral neutrophils and macrophages invade the brain tissue and degrade the myelin sheaths that protect and enhance signal transduction between neurons in the CNS^68^. While the anti-inflammatory cytokine IL-4 was also elevated, it may not be particularly effective under hypoxic conditions. The expression and secretion of these chemokines indicate that our organoid model may resemble conditions found *in vivo* in human brain diseases^69^.

Circulating cytokines, specifically IL-1α, IL-1β, IL-6, and tumor necrosis factor-α (TNF-α) can cross the BBB^35,65^. Under normal physiological conditions, low levels of cytokines interact with the luminal surface receptors of the brain microvascular endothelial cells and regulate pathways linked to cellular metabolic functions^35^. However, inflammation in a diseased organ can increase cytokine levels in the systemic circulation that in turn affect events within the brain by altering the function of the BBB. To understand the effect of circulating pro-inflammatory cytokines on the BBB, we evaluated the direct effects of IL-6 and TNF-α on the distribution of the tight junctions and adherens junction-associated proteins in our organoids. The results show that exogenous cytokines increased the disorganization of the tight junctions and increased permeability across the BBB level. This disruption was very similar to that observed in organoids cultured under hypoxic conditions. Many mechanisms may contribute to the disruption of the tight junctions. Our data show that pro-inflammatory cytokines may play an important role in disruption of the tight junctions and the dysfunction of the BBB. Even though our results show a direct effect of cytokines on BBB dysfunction, further experiments are needed to elucidate the role of hypoxia induced immunomodulation in organoids.

Given that we have shown hypoxia-induced inflammation causes BBB dysfunction, we sought to mitigate those effects. Specifically, we demonstrated that the free radical scavenger, SDG, and the endocannabinoid, 2-AG, may have protective effects against hypoxia-induced inflammation by reducing the expression of cytokines and decreasing oxidative stress that were associated with hypoxic stress conditions. Treatment with SDG and 2-AG lowered the secretion of VEGF, IL-6, and IL-8 under hypoxic condition. Results also showed maintenance of claudin-5 and beta catenin under hypoxic condition when organoids were treated with SDG and 2-AG. This could indicate that these molecules may protect BBB integrity by reducing oxidative stress and proinflammatory cytokines. However, re-oxygenated organoids had increased cytokine levels. This indicates that once inflammation is initiated, re-oxygenation may have limited effect in reducing the levels of inflammatory mediators.

The neurovascular spheroid is a suitable model for mimicking cerebral pathology, such as hypoxia, that will allow for *in vitro* testing and development of novel therapies for diseases of the central nervous system. We found that hypoxic conditions led to a change in cytokine expression and that this change in cytokine expression, such as IL-6 and TNF-α, may contribute to leakage of the BBB under hypoxic conditions. Interestingly, hypoxic conditions did not upregulate CNS MMP-2 and MMP-2 levels and hence BBB breakdown may not be due to basement membrane degradation by changes in MMP levels. Therapeutically, the free radical scavenger SDG, and the endocannabinoid, 2-AG demonstrated neuroprotective benefits with decreased expression of IL-6, IL8 and VEGF as well as increased expression of tight junction molecules Caludin-5, ZO-1 and Beta catenin. Use of 3D brain organoid models for the development of novel therapies will allow for understanding of pathologic and therapeutic mechanisms and has potential applications in CNS disease modeling.

## Methods and materials

### Cells and culture conditions

The following protocol was adapted from our previous work with minor modifications^12^. Cell expansion and differentiation protocols for all six cell types described here are similar to the methods in our previous work^12^. Primary human brain microvascular endothelial cells (Cell Systems, Kirkland, WA) were expanded in plates coated with attachment factor and were cultured under normal growth condition in complete classic medium supplemented with CultureBoostTM and attachment factor (Cell Systems). Primary human brain microvascular pericytes (HBVP; ScienCell Research Laboratories, Carlsbad, CA) were expanded in plates coated with 15 μg/ml Poly-L Lysine (ScienCell Research Laboratories) and were cultured under normal growth conditions in pericyte medium (ScienCell Research Laboratories) supplemented with 2% FBS, pericyte growth supplement and penicillin-streptomycin. Human astrocytes (HA) (ScienCell Research Laboratories) were propagated in plates coated with 0.2 mg/mL Matrigel (Corning) and were cultured under normal growth condition in astrocyte medium (ScienCell Research Laboratories) containing 2% FBS, astrocyte growth supplement, and penicillin-streptomycin. Human iPSC-derived oligodendrocyte progenitor cells (HO; Tempo Bioscience Inc., San Francisco, CA) were propagated in plates coated with 0.2 mg/mL Matrigel (Corning) and were cultured under normal growth conditions in (a) propagation media consisting of DMEM/F12 with HEPES, L- glutamine (2 mM, Life Technologies), non-Essential amino acids (1X Life Technologies), StemPro neural supplement (Invitrogen), PDGF-AA (10 ng/mL, Peprotech), PDGF-AB (10 ng/mL, Peprotech), NT3 (10 ng/mL, Peprotech), Biotin (100 ng/mL, Sigma Aldrich), and cAMP (5 μM/mL Sigma Aldrich) and were then cultured in (b) differentiation media (for 72 hrs prior to organoid formation) consisting of 50:50 DMEM/F12:neuralbasal (Life Technologies), non-essential amino acids (1X Life Technologies), 1x B27 (Life Technologies), L- glutamine (2 mM, Life Technologies), Biotin (100 ng/ml, Sigma Aldrich), PDGF-AA (5 ng/mL, Peprotech), BDNF (10 ng/ml, Peprotech), ascorbic acid (20 μg/mL, Sigma Aldrich), cAMP (1 μM/ml Sigma Aldrich), T3 (200 ng/ml, (Sigma Aldrich). Human iPSC- derived microglia (HM; Tempo Bioscience Inc., San Francisco, CA) were propagated in plates coated with 0.2 mg/ml Matrigel (Corning) and were cultured under normal growth conditions in DMEM/F-12 (Life Technologies), N2 supplement (1×, Life Technologies), essential amino acids (0.5×, Life Technologies) L-glutamine 2 mM, LifeTech), GM-CSF (100 ng/mL, Peprotech), IL-34 (50 ng/mL, Peprotech). Human iPSC- derived neural stem cells (HN) (Axol Biosciences Ltd., Cambridge, UK) were plated on plates coated with SureBond (Axol Biosciences) and cultured under normal growth conditions in 50% neural plating-XF medium (Axol Biosciences) and 50% neural expansion-XF medium (Axol Biosciences) supplemented with recombinant human FGF2 (20 ng/mL, Axol Biosciences) and recombinant human EGF (20 ng/mL, Axol Biosciences). After 48 hours, the media was then replaced with 100% neural expansion-XF medium (Axol Biosciences) supplemented with recombinant human FGF2 (20 ng/mL, Axol Biosciences) and recombinant human EGF (20 ng/mL, Axol Biosciences).

### Organoid culture

The following protocol was adapted from our previous work with minor modifications for some experiements^12^. HBMEC and HBVP were harvested using TrypLE select enzyme (1X Life Technologies), HA, HM and HO were harvested from culture plates with accutase (Life Technologies) and HN were harvested with unlock-XF (Axol Bioscience). The organoids contained 30% HBMEC, 15% HBVP, 15% HA, 5% HM, 15% HO, and 20% HN with approximately 2000 cells per organoid, except for organoids in Fig. 7A,C and Dcontained 4000 cells each. Organoids containing HA, HM, HO and HN were allowed to form in 50% astrocyte medium without astrocyte growth supplements and 50% neural maintenance-XF medium under normal growth conditions for 48 hrs using the hanging drop culture method in hanging drop culture plates (InSphero AG, Schlieren, Switzerland). The medium was mixed with heat inactivated FBS (Thermo Fisher) and 10 ng/μL rat tail collagen I (Corning). HBMEC and HBVP suspended in 50% Astrocyte media and 50% complete classic media were subsequently added to the neural-glial organoid thereby allowing the two cell types to coat the surface of the neuro-glia organoid. The organoids were cultured under normal growth conditions in 60% neural maintenance-XF medium, 20% astrocyte medium and 20% complete classic medium (Organoid Media). The organoids were then allowed to mature further for 48 hrs and were dropped into a 96 well plate for long term use. The organoids used in all subsequent studies were between 6–12 days *in vitro*.

### Cell viability

The following procedure was adapted from our previous work^12^. Cell viability was evaluated using 2 μM calcein AM (Invitrogen, green- live cells) and 4 μM ethidium homodimer-1 (Invitrogen, red - dead cells). After washing once with DPBS, the organoids were then imaged using the Olympus Fluoview Fv10i (Olympus, Tokyo, Japan) laser scanning confocal microscope.

Cell viability was evaluated using a Molecular Probe Live-Dead cell imaging system (Invitrogen).Human brain organoids were harvested for cell viability analysis at days 4, 5, 7, 10 and 21. Organoids were incubated at room temperature for 10 minutes in DPBS containing 2 μM calcein AM (Invitrogen, green- live cells) and 4 μM ethidium homodimer-1 (Invitrogen, red - dead cells). After washing once with DPBS, the organoids were then imaged using the Olympus Fluoview Fv10i (Olympus, Tokyo, Japan) laser scanning confocal microscope. The images obtained were then quantified using ImageJ to determine cell viability percentages.

### Hypoxia

On day 6, half of the 96 well plates containing the organoids were cultured in Organoid Media at 37 °C, 0.1%O_2_, 99.9%N_2_ for 24 hrs in an X*vivo* System G300C (BioSpherix, Redfield, NY, USA). Acute hypoxia *in vitro* models have been reported by others^70,71^.

### Assessment of FITC labeled IgG permeability and FITC albumin permeability

The following procedure was adapted from Nzou *et al*.^12^. Prior to incubating the organoids into the hypoxia chamber, ten organoids pre group were pooled and incubated with either FITC Conjugate IgG (1:200, Millipore) or with FITC Albumin (50 μg/mL) for 30 minutes. The organoids were washed three times before imaging with the Olympus Fluoview Fv10i (Olympus) laser scanning confocal microscope. Albumin and IgG permeability was assessed again after culturing organoids under hypoxia for 24hrs. Permeability in organoids under normoxia was also evaluated for comparison. FITC labeled albumin color was adjusted using Fluoview Fv10i image analysis software.

#### Hypoxia and oxidative stress detection

On day 7, 16 organoids were pooled into an eppendorf tube for each group and the Organoid Media was aspirated. Hypoxia/Oxidative stress detection mix was prepared by adding 5 µl of Oxidative Stress Reagent and Hypoxia Detection Reagent (Enzo Life Sciences, Farmingdale. New York) into 10 ml Organoid Medium. The organoids were then incubated with the detection mix for 30 min (37 °C at 5% CO_2_). The organoids were subsequently washed twice with PBS before fluorescent confocal imaging. The controls were prepared as per manufacturer’s instructions.

#### ATP production

The following procedure was adapted from our previous work^12^. The organoids were transferred to an opaque-walled 96 well plate. Media (100 μL) was added to wells. The reagent (100 μL) CellTiter-Glo 3D Reagent (Promega Life Sciences, Madison, Wisconsin) was then added to 100 μL of Organoid Medium containing organoids. The contents were then mixed well on an orbital shaker to induce cell lysis. The luminescent signal was stabilized by incubating the plate at room temperature for 10 minutes. Luminescence was measured using a microplate luminometer (Veritas, Mountain View, CA). Background luminescence was then subtracted from the samples.

#### Protein and cytokine quantification

Unless stated, approximately 80 organoids were pooled into Eppendorf tubes and dissociated in 25 µL Dispase I (Sigma Aldrich, St Louis, Missouri) and 475 µL RIPA buffer containing 1 mM protease and phosphatase inhibitor cocktail. The organoids were gently shaken at 37 °C for 1 hour and the cell lysates were used subsequent experiments. The BCA protein assay was performed to determine total protein concentration extracted from 80 organoids. To determine whether protein concentration is consistent in organoids, groups of 80 organoids from six randomly selected 96 well plates were pooled. Protein extraction and quantification was then performed. Given that protein levels were approximately equal in 6 randomly selected plates, reported target protein concentrations and comparisons were based on the number of organoids used in each experiment. Cell lysates were then used to determine amounts of the specific protein using ELISA. Supernatant collected from approximately 80 wells containing organoids was used to quantify cytokines levels and other soluble proteins. ELISAs were performed as per manufacturer’s instruction. Optical density for that were below the lowest standard had negative protein values. In this manuscript, the data for samples with optical density values below the lowest standard are represented as zero amount of protein. ELISA kits used: Human AQP4/Aquaporin 4, Human ABCB1/MDR1/ P-gp, Human OCLN/ Occludin, Human TJP1/ZO-1, Human CD144 /VE-Cadherin, and Human CLDN5/ Claudin 5 (Life Span Biosciences. Seattle, WA), Human SLC2A1/Glucose transporter 1 and Human COL4/ Collagen Type 4 (Abbexa. Houston, Texas), Human HSP27, HIF-1α, and HSP90α (Invitrogen. Carlsbad, CA), Human Laminin/LAMA1, Human IL2, Human MMP-2, Human VEGF, Human IL4, Human MMP-9, Human IL10, Human TNF-α, Human IL-8, Human IL6, Human MCP-1, Human IL-1β, and Human FN1/Fibronectin (BosterBio. Pleasanton, CA).

#### Secoisolariciresinol diglycoside (SDG) and 2-Arachidonyl glycerol (2-AG) treatments

Organoids in Organoid Media were treated under normal culture conditions (**Normoxia**). Two separate groups of 80 organoids were cultured in Organoid Media containing secoisolariciresinol diglycoside, (50 µM, Sigma) or 2-arachidonyl glycerol (10 µM, Sigma) for 48 hours prior to culturing under hypoxic condition (*). Organoid Media was then replaced with a fresh full media change containing secoisolariciresinol diglycoside or 2-arachidonyl glycerol before incubating in hypoxic chamber for 24 hours. Another group of 80 organoids were treated during hypoxic exposure only (**#**), Two groups of 80 organoids each were cultured under hypoxic conditions without Secoisolariciresinol diglycoside or 2-Arachidonyl glycerol (**Hypoxia**) and one of which was re-oxygenated under normoxic conditions for 24 hours (**HR)**. All groups were subsequently stored at −80 °C. The organoids and supernatants were then collected, and the cytokines were assessed from the supernatants using ELISA while the organoids were fixed for immunohistochemistry.

##### Immunohistochemistry

The following procedure was adapted from our previous work^12^. Unless stated, about 10–20 organoids were collected into 1.7 ml Eppendorf tubes (Corning Inc). After aspirating the media, the organoids were fixed in 4% formaldehyde (Polysciences Inc, Warrington, PA) for 15 minutes at 4 °C and were washed 3 times with cold PBS. The organoids were permeabilized with 0.1% Tween-20 in PBS for 10 minutes at 4 °C and washed 3 times. Organoids were exposed to Protein Block (Dako Group, Troy, MI) for 1 hr at RT, and organoids incubated at 4 °C overnight in Antibody Diluent (Dako) solution containing primary antibodies, anti-beta Catenin (1:500, Abcam), anti-ZO-1 (1:1000, Millipore), and anti-Claudin-5 (1:500, Millipore). Organoids were subsequently washed 3 times and incubated with AF488 Goat anti-Mouse IgG (1:1000, Life Technologies), AF594 Goat Anti-Rabbit IgG (1:1000, Life Technologies) in Antibody diluent (Dako) overnight at 4^o^C. Nuclear staining was performed by incubating the organoids with DAPI (1:1000) in PBS for 10 minutes. The organoids were washed and were imaged using the Olympus Fluoview Fv10i (Olympus) laser scanning confocal microscope. Unless stated, at least three randomly selected organoids were imaged for each stain.

## Data Availability

All data generated or analyzed during this study are included in this article.
