# Author Correction: Multicellular 3D Neurovascular Unit Model for Assessing Hypoxia and Neuroinflammation Induced Blood-Brain Barrier Dysfunction

**DOI:** 10.1038/s41598-020-77348-9

**Published:** 2020-11-18

**Authors:** Goodwell Nzou, Robert T. Wicks, Nicole R. VanOstrand, Gehad A. Mekky, Stephanie A. Seale, Aya EL-Taibany, Elizabeth E. Wicks, Carl M. Nechtman, Eric J. Marrotte, Vishruti S. Makani, Sean V. Murphy, M. C. Seeds, John D. Jackson, Anthony J. Atala

**Affiliations:** 1 Wake Forest Institute for Regenerative Medicine, Wake Forest School of Medicine, Winston-Salem, NC 27101 USA; 2 Department of Neurology and Neurological Surgery, Wake Forest Baptist Medical Center, Winston-Salem, NC 27157 USA; 3 Zoology Department, Faculty of Science, Zagazig University, Zagazig, Egypt

Correction to: *Scientific Reports* 10.1038/s41598-020-66487-8, published online 17 June 2020

The original version of this Article contained a typographical error in the spelling of the author Eric J. Marrotte, which was incorrectly given as Eric J. Marotte. This has now been corrected in the PDF and HTML versions of the Article.

